# Polymyalgia Rheumatica After ChAdOx1 nCov-19 Vaccine: A Case Report

**DOI:** 10.7759/cureus.25346

**Published:** 2022-05-26

**Authors:** Carolina Lourenço, Adriana Pascoal, António Paiva, Inês Campos, José Pagaimo

**Affiliations:** 1 Physical Medicine and Rehabilitation, Centro de Medicina de Reabilitação da Região Centro - Rovisco Pais, Tocha, PRT; 2 Internal Medicine, Centro de Medicina de Reabilitação da Região Centro - Rovisco Pais, Tocha, PRT

**Keywords:** reactive arthritis, inflammatory musculoskeletal manifestations, chadox1 vaccine, covid-19 vaccine, polymyalgia rheumatica

## Abstract

Polymyalgia rheumatica (PMR) is an inflammatory rheumatic disease characterized by severe pain and morning stiffness, mainly affecting the shoulder girdle.

A 75-year-old woman, previously healthy, received the first dose of ChAdOx1 vaccine and two weeks later started with pain in the shoulder and pelvic girdles and knees of inflammatory characteristics, accompanied by morning stiffness (about one hour), anorexia, asthenia, and activities of daily living (ADL) dependence. She started analgesics and non-steroidal anti-inflammatory drugs (NSAIDs) with no improvement. The symptoms aggravated three days after the second vaccine dose, and she was referred to our center. At observation, she presented shoulder, hip, and knee active range of motion limitation. Blood analysis revealed an Erythrocyte Sedimentation Rate (ESR) of 120mm/h (reference value < 20mm/h) and C-Reactive Protein (CRP) of 80mg/L (reference value < 5mg/L). Ultrasound showed effusion on both shoulders, hips, and knees. The paraneoplastic syndrome was ruled out.

She started oral corticosteroids and a rehabilitation program, and a month later, she presented controlled pain, normal analysis, and ADL independence.

This case shows symptomatic and analytic features of PRM after the first vaccine dose and aggravation soon after the second. As such, we consider establishing a potential relationship between the inoculation and the development of PRM.

A few cases were published reporting a PRM-like syndrome following a COVID-19 vaccine; however, the underlying mechanism and prognosis are still unknown.

## Introduction

Polymyalgia rheumatica (PMR) is an inflammatory rheumatic condition characterized by severe pain and morning stiffness (about one hour) affecting the shoulder girdle and proximal aspects of the arms bilaterally. Pain and stiffness are also common in the neck, pelvic girdle, and the proximal aspects of the thighs [1].

PMR is the second most common systemic rheumatic disease in adults, with an estimated lifetime risk of 2.4% for women and 1.7% for men, with more than two-thirds of the patients being women. It’s rare under the age of 50 and has its peak incidence in the 70-79 age group [2].

PMR pathophysiology is poorly understood. However, the higher incidence in older people suggests that age may be a contributing factor in addition to a state of inflammation translated by the increase of acute phase reactants (C-reactive protein [CRP] and erythrocyte sedimentation rate [ESR]), which is a strong indicator for diagnosis [3].

There are no established diagnostic criteria for PMR, so the following criteria are used (when no other pathology explains the findings): age of 50 years or older, bilateral shoulder or pelvic girdle aching, or both for more than two weeks and lasting more than 45 minutes with morning stiffness, the elevation of acute phase reactants (CRP and ESR), absence of rheumatoid factor and anti-citrullinated peptide antibodies and rapid symptom resolution with low-dose glucocorticoids (15-25mg/day of prednisolone or equivalent). If ultrasound is available, the hip or shoulder bursitis and biceps tenosynovitis reinforce the diagnosis hypothesis [1,4].

Also present in about 20% of PMR patients, is giant cell arteritis, a large blood vessel vasculitis presenting usually as a new-onset headache, scalp tenderness, and jaw claudication. Due to this high prevalence association, giant cell arteritis must always be excluded in PMR patients [5].

## Case presentation

A 75-year-old woman, previously asymptomatic and without relevant personal or familiar background, presented with bilateral shoulder and hip pain that lasted for about four months. She received the first dose of the ChAdOx1 vaccine on April 15^th^, 2021, with no significant adverse effects. Two weeks later, she started complaining about generalized pain, mostly in the shoulder and pelvic girdles, mainly in the morning and during the night, at awakening the patient experienced the pain; she had morning stiffness (for about one hour), anorexia, and asthenia that conditioned ADL dependence and hindered her in attending her gymnastics class. There was no history of fever. Three days later, she went to her family doctor and was tested for SARS-CoV 2 (negative) and was medicated with an association of tramadol 37.5mg and paracetamol 325mg, and an NSAID. She underwent hip and shoulder radiographs (that revealed slight degenerative alterations) and a shoulder ultrasound that showed periarticular inflammation. Two weeks later, she presented no improvement and was prescribed a single dose of intramuscular betamethasone 14mg/2mL. After that, she started slowly improving in the weeks following.

On July 8^th^, 2021, she received the second ChAdOx1 vaccine dose, and three days later, the previous complaints returned with the same characteristics. A month later, she repeated a single dose of intramuscular betamethasone 14mg/2mL and was referred to our Physical and Rehabilitation Medicine consultation.

The patient was first observed on August 11^th^, 2021, and presented with limited active range of motion of both shoulders (particularly lateral and anterior elevations, limited to about 80o; and internal rotation bilaterally) and hips and pain in all the specific shoulder tests, including conflict; no other relevant alterations were found on examination. We requested blood analysis that revealed normocytic and normochromic anemia, an ESR of 120mm/1h (reference value < 20mm/1h), and CRP of 80mg/L (reference value < 5mg/L); auto-antibodies (anticitrullinated peptide antibodies, rheumatoid factor, antinuclear antibodies, and anti-neutrophil cytoplasmic antibodies) were negative. Ultrasound evaluation revealed effusion on both shoulders, hips, and knees. After that, she started prednisolone 20mg id on September 24^th^ with calcium and vitamin D supplementation for osteoporosis prophylaxis. A rehabilitation program was also prescribed for pain relief, range of motion improvement, and muscle strengthening. The complementary study was performed with an endoscopic procedure, a colonoscopy, a thoracoabdominal-pelvic CT scan, and breast and thyroid ultrasound and excluded the possibility of a neoplasm because a paraneoplastic syndrome could mimic the aforementioned presentation.

About a month later, on October 27^th ^2021, she was re-evaluated and was significantly improved since the first days of treatment, with no morning pain or stiffness. Physical examination presented only a slight shoulder internal rotation limitation. Blood tests revealed normal ESR and CRP. Functionally she regained independence but had some difficulties in some instrumental ADL (like cleaning and doing laundry) and hadn’t resumed gymnastics.

In December, we started prednisolone. Currently, eight months after the beginning of treatment, she is taking 12.5 mg, and no relapse was detected.

## Discussion

COVID-19 vaccines have been administrated to billions of people all over the world. We currently have mRNA-based vaccines (that include BNT 162b2 and mRNA vaccines) and adenoviral vector-based (ChAdOx1 and Ad26.COV2 vaccines).

The adverse effects of COVID-19 vaccines are usually mild and located at the administration site [6]. Although arthralgia is a common adverse effect, reactive arthritis is rather rare, and only a few cases were reported. A recently published case series with 66 patients with inflammatory musculoskeletal manifestations (e.g., synovitis, tenosynovitis, enthesitis, inflammatory spinal pain, or girdles pain/stiffness with serological evidence of inflammation) with onset within four weeks from the administration of one of the COVID-19 vaccines revealed that findings resembling polymyalgia rheumatica (PMR-like) were the most common with 27 cases reported; 18 of them after the BNT162b2 vaccine but no pathology was associated with a specific vaccine type [7].

Current pathogenesis for vaccine-associated autoimmunity is attributed to cross-reactivity between antigens or the adjuvant effect. PMR has already been thought to be in the spectrum of autoimmune syndromes induced by adjuvants, and a self-limited form of PMR has previously been associated with influenza vaccines, particularly in persons with a higher spontaneous risk, such as those with a family history [8].

A few cases were published reporting a PMR-like syndrome following a COVID-19 vaccine similar to our case; however, the underlying mechanism is still to be found [9-11]. The prognosis of these patients seems favorable since most of the cases report a self-limiting pathology; nevertheless, there is still insufficient data [7].

## Conclusions

This case shows symptomatic and analytic features of PMR after the first vaccine dose and aggravation soon after the second. As such, we consider that a potential relationship between inoculation and the development of PRM can be established. Our patient responded well to corticosteroid therapy and the adjuvant rehabilitation program resuming her independence in ADL. Similar reactions had already been described with some other inflammatory musculoskeletal manifestations and, therefore, should be suspected after vaccine inoculation. Even assuming this direct relationship, the authors are still unanimously affirming that the vaccine's benefits far outweigh the risk.

## References

[1] González-Gay MA, Matteson EL, Castañeda S (2017). Polymyalgia rheumatica. Lancet.

[2] Crowson CS, Matteson EL, Myasoedova E (2011). The lifetime risk of adult-onset rheumatoid arthritis and other inflammatory autoimmune rheumatic diseases. Arthritis Rheum.

[3] Carvajal Alegria G, Boukhlal S, Cornec D, Devauchelle-Pensec V (2020). The pathophysiology of polymyalgia rheumatica, small pieces of a big puzzle. Autoimmun Rev.

[4] Dasgupta B, Borg FA, Hassan N (2010). BSR and BHPR guidelines for the management of polymyalgia rheumatica. Rheumatology (Oxford).

[5] Younger DS (2019). Giant cell arteritis. Neurol Clin.

[6] Pormohammad A, Zarei M, Ghorbani S, Mohammadi M, Razizadeh MH, Turner DL, Turner RJ (2021). Efficacy and safety of COVID-19 vaccines: a systematic review and meta-analysis of randomized clinical trials. Vaccines (Basel).

[7] Ursini F, Ruscitti P, Raimondo V (2022). Spectrum of short-term inflammatory musculoskeletal manifestations after COVID-19 vaccine administration: a report of 66 cases. Ann Rheum Dis.

[8] Liozon E, Parreau S, Filloux M (2021). Giant cell arteritis or polymyalgia rheumatica after influenza vaccination: a study of 12 patients and a literature review. Autoimmun Rev.

[9] Chan JE, Irimpen A (2021). Severe but self-limiting polyarthralgia with functional impairment following ChAdOx1 nCov-19 vaccination in an elderly recipient. Vaccines (Basel).

[10] Osada A, Sakuragi C, Toya C, Mitsuo A (2022). New-onset polymyalgia rheumatica following the administration of the Pfizer-BioNTech COVID-19 vaccine. Intern Med.

[11] Izuka S, Komai T, Natsumoto B, Shoda H, Fujio K (2022). Self-limited polymyalgia rheumatica-like syndrome following mRNA-1273 SARS-CoV-2 vaccination. Intern Med.

